# β-arrestin 2 Is a Prognostic Factor for Survival of Ovarian Cancer Patients Upregulating Cell Proliferation

**DOI:** 10.3389/fendo.2020.554733

**Published:** 2020-09-18

**Authors:** Bastian Czogalla, Alexandra Partenheimer, Udo Jeschke, Viktoria von Schönfeldt, Doris Mayr, Sven Mahner, Alexander Burges, Manuela Simoni, Beatrice Melli, Riccardo Benevelli, Sara Bertini, Livio Casarini, Fabian Trillsch

**Affiliations:** ^1^Department of Obstetrics and Gynecology, University Hospital, LMU Munich, Munich, Germany; ^2^Department of Obstetrics and Gynecology, University Hospital Augsburg, Augsburg, Germany; ^3^Institute of Pathology, Faculty of Medicine, LMU Munich, Munich, Germany; ^4^Unit of Endocrinology, Department of Biomedical, Metabolic and Neural Sciences, University of Modena and Reggio Emilia, Modena, Italy; ^5^Center for Genomic Research, University of Modena and Reggio Emilia, Modena, Italy; ^6^Unit of Endocrinology, Department of Medical Specialties, Azienda Ospedaliero-Universitaria, Modena, Italy; ^7^PRC, INRA, CNRS, IFCE, Université de Tours, Nouzilly, France

**Keywords:** β-arrestin 2, ovarian cancer, immunohistochemistry, survival analysis, G protein-coupled receptor, *in vitro* analyses

## Abstract

Establishing reliable prognostic factors as well as specific targets for new therapeutic approaches is an urgent requirement in advanced ovarian cancer. For several tumor entities, the ubiquitously spread scaffold protein β-arrestin 2, a multifunctional scaffold protein regulating signal transduction and internalization of activated G protein-coupled receptors (GPCRs), has been considered with rising interest for carcinogenesis. Therefore, we aimed to elucidate the prognostic impact of β-arrestin 2 and its functional role in ovarian cancer. β-arrestin 2 expression was analyzed in a subset of 156 samples of ovarian cancer patients by immunohistochemistry. Cytoplasmic expression levels were correlated with clinical as well as pathological characteristics and with prognosis. The biologic impact of β-arrestin 2 on cell proliferation and survival was evaluated, *in vitro*. Following transient transfection by increasing concentrations of plasmid encoding β-arrestin 2, different cell lines were evaluated in cell viability and death. β-arrestin 2 was detected in all histological ovarian cancer subtypes with highest intensity in clear cell histology. High β-arrestin 2 expression levels correlated with high-grade serous histology and the expression of the gonadotropin receptors FSHR and LHCGR, as well as the membrane estrogen receptor GPER and hCGβ. Higher cytoplasmic β-arrestin 2 expression was associated with a significantly impaired prognosis (median 29.88 vs. 50.64 months; *P* = 0.025). Clinical data were confirmed in transfected HEK293 cells, human immortalized granulosa cell line (hGL5) and the ovarian cancer cell line A2780 *in vitro*, where the induction of β-arrestin 2 cDNA expression enhanced cell viability, while the depletion of the molecule by siRNA resulted in cell death. Reflecting the role of β-arrestin 2 in modulating GPCR-induced proliferative and anti-apoptotic signals, we propose β-arrestin 2 as an important prognostic factor and also as a promising target for new therapeutic approaches in advanced ovarian cancer.

## Introduction

Epithelial ovarian cancer (EOC) is the most lethal gynecological malignancy and is considered the fifth leading cause of death among women (1), mainly due to late diagnosis in advanced tumor stage leading to overall poor prognosis. Lack of inadequate screening methods and rising resistances toward chemotherapy over the clinical course further contribute to the relatively low 5-years survival at around 45% (1, 2). Standard treatment for advanced EOC consists of primary cytoreductive surgery, followed by platinum-based combination chemotherapy followed by targeted therapies like the anti-angiogenic antibody bevacizumab or PARP inhibitors. To date, the most reliable prognostic factors include volume of post-operative residual disease, tumor stage diagnosed according to the International Federation of Gynecology and Obstetrics (FIGO) staging system, patient's age and histology (2–7). EOCs are classified into four histological subtypes: serous, mucinous, endometrioid, and clear-cell, which vary in terms of phenotype, etiology and molecular background (8). Taking the heterogeneity of ovarian cancer into account appears crucial for developing new prognostic and therapeutic strategies.

β-arrestin 2 is a multifunctional scaffold protein regulating signal transduction and internalization of activated G protein-coupled receptors (GPCRs) like follicle-stimulating hormone receptor (FSHR) and luteinizing hormone/choriogonadotropin receptor (LHCGR) by promoting clathrin-dependent endocytosis (9). The β-arrestin family is a known activator of signaling pathways involved in tumorgenesis (10–12) and proliferation of certain tumor entities (13–17), including ovarian cancer (18). Functional association between FSHR/LHCGR and β-arrestin in granulosa cells could be demonstrated after gonadotropin treatment *in vitro* (19, 20). In immortalized ovarian cell lines, β-arrestin 2 triggers the activation of FSH-induced mitogen-activated protein (MAP) kinase signaling cascades linked to anti-apoptotic and proliferative events (21).

In this study, we aimed to evaluate the possible prognostic impact of β-arrestin 2 on clinical course of ovarian cancer. The role of β-arrestin 2 in modulating cell viability and survival was elucidated by functional analyses *in vitro*, suggesting new therapeutic approaches in ovarian cancer.

## Materials and Methods

### Patients and Specimens

156 ovarian cancer samples of patients that underwent surgery between 1990 and 2002 at the Department of Obstetrics and Gynecology, Ludwig Maximilian University in Munich, Germany, were analyzed (Table 1). The clinical data were received from the patient's charts, whereas the follow-up data from the Munich Cancer Registry (MCR). Only patients with a clear diagnosis of ovarian cancer and were included in this study, while benign tumors, just as borderline tumors were excluded. These patients had no history of previous or secondary cancers. Also, none of the patients had neoadjuvant chemotherapy. After the samples had been formalin-fixed and paraffin-embedded (FFPE), they were evaluated by a specialized pathologist at the department of Pathology, Ludwig Maximilian University, who classified them into histological subtypes [serous (*n* = 110), endometrioid (*n* = 21), mucinous (*n* = 13), clear-cell (*n* = 12)] and rated the tumor grading. The serous ovarian cancer samples were divided into low and high grading. Endometrioid ovarian cancer was graded according to G1–G3. For the mucinous carcinoma, there is no WHO classification; however, the subtype is often classified into G1–G3. The clear cell cancer was always categorized as G3. Staging was performed using the FIGO (WHO) and TNM classification. I (*n* = 35), II (*n* = 10,) III (*n* = 103), and IV (*n* = 3). Data on primary tumor extension was available in 155 cases: T1 (*n* = 40), T2 (*n* = 18), T3 (*n* = 93), T4 (*n* = 4), and on lymph node involvement in 95 cases: N0 (*n* = 43), N1 (*n* = 52). In nine cases available was the data on distant metastasis: M0 (*n* = 3), M1 (*n* = 6).

**Table 1:** Patient characteristics.

**Parameters**	***N***	**Percentage (%)**
**HISTOLOGY**		
Serous	110	70.5
Clear cell	12	7.7
Endometrioid	21	13.5
Mucinous	13	8.3
**LYMPH NODE**		
pNX	61	39.1
pN0	43	27.6
pN1	52	33.3
**DISTANT METASTASIS**		
pM0/X	150	96.2
pM1	6	3.8
**GRADING SEROUS**		
Low	24	23.0
High	80	77.0
**ENDOMETRIOID**		
G1	6	31.6
G2	5	26.3
G3	8	42.1
**MUCINOUS**		
G1	6	50.0
G2	6	50.0
G3	0	0
**CLEAR CELL**		
G3	9	100
**FIGO**		
I	35	23.1
II	10	6.6
III	103	68.2
IV	3	2.0
**AGE**		
≤ 60 years	83	53.2
>60 years	73	46.8

### Ethical Approval

This study was approved by the Ethics Committee of the Ludwig-Maximilians-University, Munich, Germany (approval number 227-09 and 18-392). All tissue samples used for this study were obtained from leftover material from the archives of LMU Munich, Department Gynecology and Obstetrics, Ludwig-Maximilians-University, Munich, Germany, initially used for pathological diagnostics. The diagnostic procedures were completed before the current study was performed. During all experimental and statistical analysis, the observers were fully blinded to patient's data. All experiments were performed according to the standards of the Declaration of Helsinki (1975). As per declaration of our ethics committee no written informed consent of the participants or permission to publish is needed given the circumstances described above.

### Immunohistochemistry

The formalin-fixed and paraffin-embedded ovarian cancer tissue samples were dewaxed in xylene for 20 min, before adding 100% ethanol to completely remove the Xylol. Unspecific color responses were avoided by blocking the endogenous peroxidase with 3% H_2_O_2_ in methanol (3 ml 30% H_2_O_2_ + 97 ml methanol), followed by rehydrating it in 70%, then 50% ethanol. Afterwards the slides were placed in a pressure cooker for 10 min, using sodium citrate buffer (pH = 6), made from 0.1 M citric acid and 0.1 M sodium citrate). After cooling down, this was followed by washing the samples first in distilled water, then in phosphate buffered saline (PBS) twice. After preprocessing, the slides were incubated in a blocking solution (ZytoChem Plus HRP Polymer System, Berlin, Germany, POLHRP-100) for 5 min to prevent an unspecific staining reaction. This was followed by a 16-h incubation overnight at 4°C with the primary antibodies: anti-β-arrestin 2, rabbit IgG, polyclonal, Abcam, ab151774 at a 1:1,500 dilution; anti-FSHR, rabbit IgG, polyclonal, Novus Biologicals, NLS2231 at a 1:100 dilution, anti-LHCGR, rabbit IgG, polyclonal, Acris, SP4594P at a 1:800 dilution; anti-GPER, rabbit IgG, polyclonal, Lifespan Biosciences, LS92467 at a 1:600 dilution and anti-hCGβ, rabbit IgG, polyclonal, Dako, A-0231 at a 1:300 dilution—then washed in PBS twice. Next step was the application of reagent 2 (ZytoChem Plus HRP Polymer System, Berlin, Germany, POLHRP-100), consisting of a corresponding biotinylated secondary anti-rabbit IgG antibody and the associated avidin-biotin-peroxidase complex, for 20 min. For the visualization reaction 3,3 Diaminobenzidine (DAB) and a substrate buffer (Liquid DAB and Substrate Chromogen System, Dako, Munich, Germany, K3468) was used for 30 min, followed by distilled water, to stop the reaction. After counterstaining the slides with Mayer's acidic hematoxylin (Waldeck-Chroma, Münster, Germany, catalog-number 2E-038) for 2 min, they were dehydrated in an ascending series of alcohol (70, 96, 100%), brightened by adding xylol and then covered. Negative and positive controls were used to assess the specificity of the immunoreactions. Negative controls (colored in blue) were performed in kidney, placental and uterine tissue by replacement of the primary antibodies by species-specific (rabbit) isotype control antibodies (Dako, Glostrup, Denmark). For positive controls, placental and kidney tissues were used.

### Staining Evaluation

**Figure 1:**
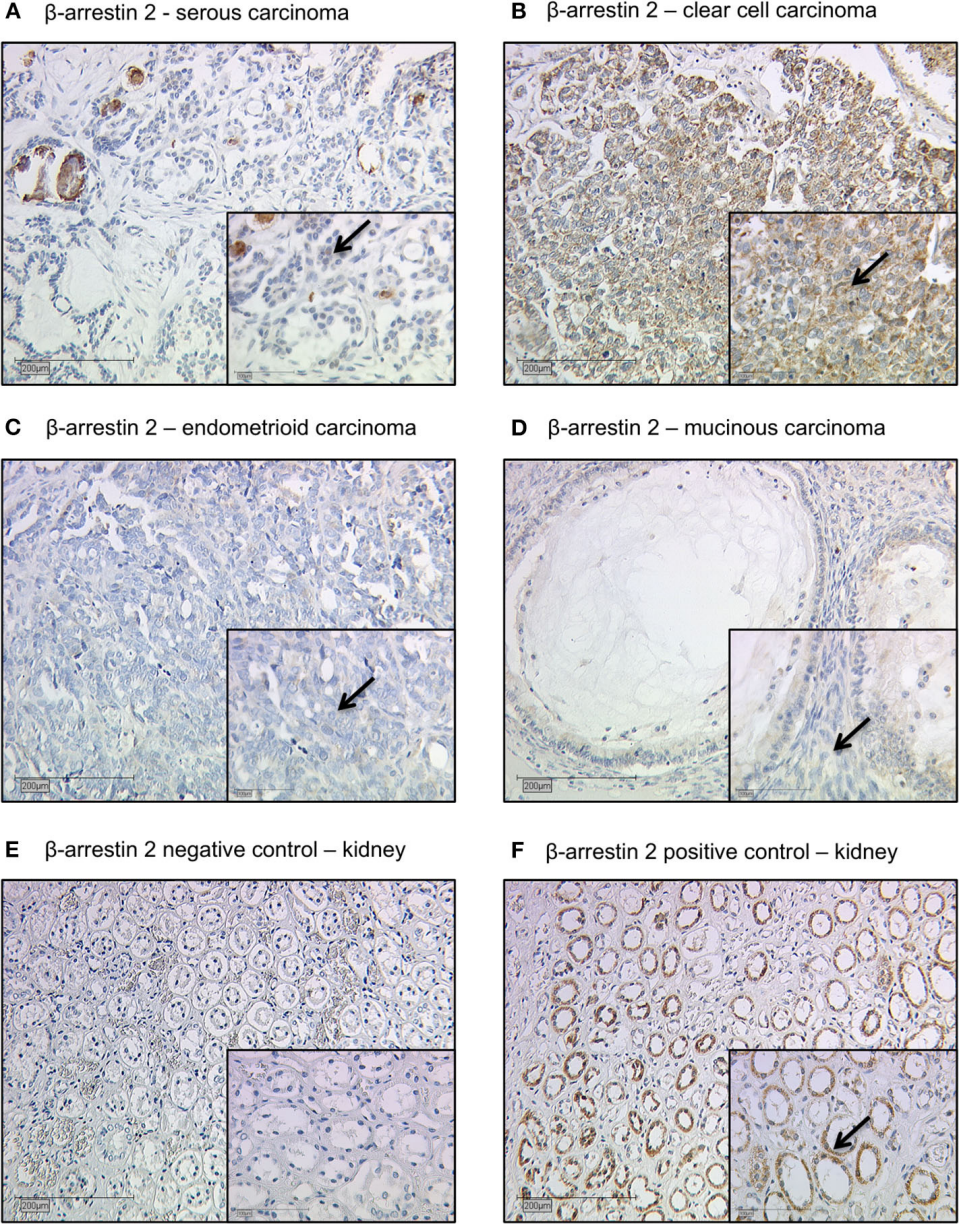
Detection of β-arrestin 2 with immunohistochemistry. **(A)** Cytoplasmic β-arrestin 2 staining in ovarian cancer with serous, **(B)** clear cell, **(C)** endometrioid, and **(D)** mucinous histology. **(E)** β-arrestin 2 negative control **(F)** and positive control in human kidney tissue. Clear cell histology exhibited the strongest cytoplasmic β-arrestin 2 expression (Kruskal-Wallis test; *p* = 0.01) when compared with different histological subtypes. Arrows demonstrate representatively β-arrestin 2 stained cells. Images representative of three independent experiments.

All EOC specimens were examined with a Leitz photomicroscope (Wetzlar, Germany) and specific β-arrestin 2 immunohistochemically staining reaction was observed in the cytoplasm of the cells. The intensity and distribution pattern of β-arrestin 2 staining was rated using the semi-quantitative immunoreactive score (IR score, Remmele's score). To obtain the IR score result, the optional staining intensity (0 = no, 1 = weak, 2 = moderate, and 3 = strong staining) and the percentage of positive stained cells (0 = no staining, 1 = <10% of the cells, 2 = 11–50% of the cells, 3 = 51–80% of the cells and 4 = >81%) were multiplied. Cut-off points for the IR scores were selected for the β-arrestin 2 staining considering the distribution pattern of IR scores in the collective. β-arrestin 2 staining was regarded as low with IRS 0–3 and as high with IRS ≥ 4. Renal biopsies from patients with different *ARRB2* expression levels served as positive and negative control staining (22) (Figures 1E,F).

### Cell Lines and Transfection Protocol

The immortalized human granulosa (hGL5) cell line of ovarian origin (23), the human embryonic kidney (HEK293) and the ovarian carcinoma (A2780) cell lines were used for *in vitro* experiments. Cells were cultured and handled as previously described (21, 24–26). hGL5 cells culture medium was DMEM/F12 supplemented with 10% fetal bovine serum (FBS) and 2 mM L-glutamine (Sigma-Aldrich, St. Louis, MO, USA), 100 IU/mL penicillin and 50 μg/mL streptomycin (Thermo Fisher Scientific, Waltham, MA, USA) and 2% ITS + Premix Universal Culture Supplement (#354352; Corning Incorporated, Corning, NY, USA). The HEK293 cell line was cultured in DMEM additioned of 10% FBS, 2 mM L-glutamine, 100 IU/mL penicillin and 50 μg/mL streptomycin. The A2780 cell line served as a model of ovarian cancer and was cultured in RPMI1640 medium (Sigma-Aldrich) additioned of 10% FBS, 1% Hepes (Sigma-Aldrich), 2 mM L-glutamine, 100 IU/mL penicillin and 50 μg/mL streptomycin. Cell lines were maintained in an incubator at 37°C and 5% CO_2_.

The transfection protocol was described previously (25–28). Briefly, 3.5 × 10^4^ cells were transiently transfected in a 96-well plate using increasing amount (range: 0–200 ng/well) of β-arrestin 2-encoding pcDNA3.1 plasmid (29) or commercial siRNA probes against β-arrestin 2 (#14387; Life-Technologies, Carlsbad, CA, USA) (21), using an electroporator (Gene Pulser MXcell; Bio-Rad Laboratories Inc., Hercules, CA, USA) in accordance with established protocol and settings (21, 30). Control probes without specific target mRNAs were loaded for achieving equal amount of siRNA among wells.

### Western Blot Analysis and Antibodies

Overexpression and knock-down of β-arrestin 2 and pro-caspase 3 were evaluated by Western blotting, as previously described (21, 24). Briefly, transfected cells were seeded in 24-well plates (1 × 10^5^ cells/well) and cultured 48 h before to be lysed using 4°C RIPA buffer. β-arrestin 2 and pro-caspase 3 were evaluated by 12% SDS-page and Western blotting, using specific antibodies (#3857, Cell Signaling Technology Inc., Danvers, MA, USA; #MA1-91637, Thermo-Fisher Scientific, respectively). β-actin signals served as loading controls and were detected using a horseradish peroxidase (HRP)-conjugated specific antibody (#A3854; Sigma-Aldrich). Signals were revealed by ECL chemiluminescent compound (GE HealthCare), after incubation of the membranes with a secondary anti-rabbit HRP-conjugated antibody (#NA9340V; GE HealthCare), except for β-actin. Western blotting signals were acquired by the Quantity One analysis software (Bio-Rad Laboratories Inc., Hercules, CA, USA).

### Analysis of Cell Viability and Death

hGLC, HEK293, and A2780 cells were seeded in 96-well plates (1 × 10^4^ cells/well) before viability assessment the by MTT colorimetric assay (31), using a previously validated protocol (21, 30). The absorbance was detected by a Victor3 plate reader (Perkin Elmer Inc., Waltham, MA, USA). A measure of death cells was obtained by incubating samples 30 min with propidium iodide (32). Cells were washed twice by PBS before excitation and light emission detection by a CLARIOstar microplate reader (BMG LabTech, Ortenberg, Germany), following the manufacturer's protocol. Data were represented as box and whiskers plots in a graph, as a measure of cell viability, and control of protein amount per well was performed by Bradford assay.

### Statistical Analysis

IBM SPSS Statistics, version 25.0 (IBM Corporation, Armonk, NY, USA) was used for collecting and analyzing all the data for statistical significance. Overall-survival was compared with Kaplan-Meier curves and log-rank testing was used to detect differences in patients' overall survival times. The Spearman correlation coefficient (cc) was used for detecting correlations between immunohistochemical staining outcomes. For multivariate analyses, the cox regression was performed. Results obtained from cell lines were analyzed by D'Agostino and Pearson normality test before applying the Kruskal-Wallis test together with Dunn's correction for multiple comparisons, using the GraphPad Prism 6.0 software (GraphPad Software Inc., La Jolla, CA, USA). For all analysis a *P*-value of < 0.05 was considered to be statistically significant.

## Results

### β-arrestin 2 Expression and the Correlation to Clinical and Pathological Characteristics

The clinicopathologic characteristics of the analyzed ovarian cancer patients are listed in Table 1. 104 (67%) out of 156 ovarian cancer specimens showed positive cytoplasmic β-arrestin 2 expression. The median immunoreactive score (IRS) of cytoplasm staining was 2 (with a range of 0–12). Clear cell histology exhibited the strongest β-arrestin 2 staining (Kruskal-Wallis test; *p* = 0.01) when compared with different histological subtypes (Figures 1A–D).

**Figure 2:**
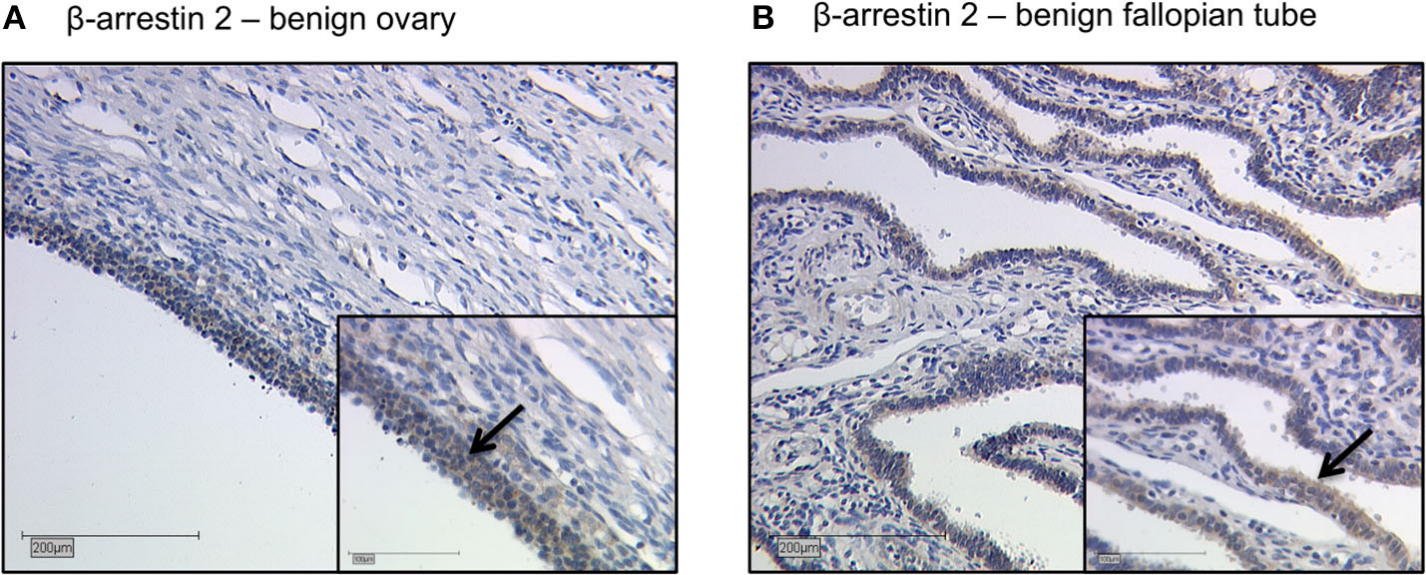
Detection of β-arrestin 2 with immunohistochemistry in benign ovary and fallopian tube. Detection of β-arrestin 2 with immunohistochemistry in benign ovary **(A)** and fallopian tube **(B)**. Positive β-arrestin 2 staining was observed in epithelial cells, granulosa cells and theca cells. The ovarian and fallopian tube stroma did not show a β-arrestin 2 staining. Arrows demonstrate representatively β-arrestin 2 stained cells. Images representative of three independent experiments.

Representative staining of benign ovarian and fallopian tube tissue show positive results in epithelial cells, granulosa cells and theca cells. The ovarian and fallopian tube stroma did not show a β-arrestin 2 staining (Figures 2A,B).

We analyzed the correlation between β-arrestin 2 and clinicopathological data, such as histology, grading, and FIGO classification (Table 2). A positive correlation was observed between high β-arrestin 2 expression and high-grade serous histology (Spearman's correlation analysis; *p* = 0.041; *cc* = 0.169). Moreover, no other correlations were observed.

**Table 2:** Correlation analysis between β-arrestin 2 expression and clinicopathological data.

**Variable**	***P***	**Correlation coefficient**
Histology	0.075	−0.083
FIGO stage	0.673	0.086
Age	0.206	0.105
**GRADING**		
Clear cell, endometrioid, and mucinous (G1–G3)	0.520	−0.088
Serous—low grading	0.052	−0.160
Serous—high grading	0.041^*^	0.169

Clinicopathologic data and β-arrestin 2 expresssion were correlated to each other using Spearman's correlation analysis. Significant correlations are indicated by asterisks

(* *p < 0.05). p, two-tailed significance*.

### β-arrestin 2 Correlates With Gonadotropin Receptors Expression

We then analyzed the correlation between β-arrestin 2 expression and other parameters previously examined (33–37) in the same ovarian cancer cohort (Table 3). A positive correlation was detected to the expression of the gonadotropin receptors FSHR (*p* = 0.029; *cc* = 0.183) and LHCGR (*p* = 0.001; *cc* = 0.284), the G protein-coupled membrane estrogen receptor GPER (*p* = 0.0009; *cc* = 0.215) as well as with the choriogonadotropin beta subunit (hCGβ) (*p* < 0.001; *cc* = 0.36). Representative stainings of the correlated parameters are shown in Supplementary Figure 1.

**Table 3:** Correlation analysis of β-arrestin 2.

**Staining**	**β-arrestin 2**	**FSHR**	**LHCGR**	**GPER**	**hCGβ**
**β-ARRESTIN 2**					
*cc*	1.000	0.183	0.284	0.215	0.360
*p*	–	0.029	0.001	0.009	0.000
*n*	148	143	146	148	137
**FSHR**					
*cc*	0.183	1.000	0.164	0.214	0.082
*p*	0.029	–	0.046	0.009	0.340
*n*	143	151	149	148	137
**LHCGR**					
*cc*	0.284	0.164	1.000	0.170	0.200
*p*	0.001	0.046	–	0.037	0.018
*n*	146	149	154	151	140
**GPER**					
*cc*	0.215	0.214	0.170	1.000	0.114
*p*	0.009	0.009	0.037	–	0.179
*n*	148	148	151	153	141
**hCGβ**					
*cc*	0.360	0.082	0.200	0.114	1.000
*p*	0.000	0.340	0.018	0.179	–
*n*	137	137	140	141	142

*cc, correlation coefficient; p, two-tailed significance; n, number of patients*.

### High β-arrestin 2 Expression Is Associated With Impaired Overall Survival

**Figure 3:**
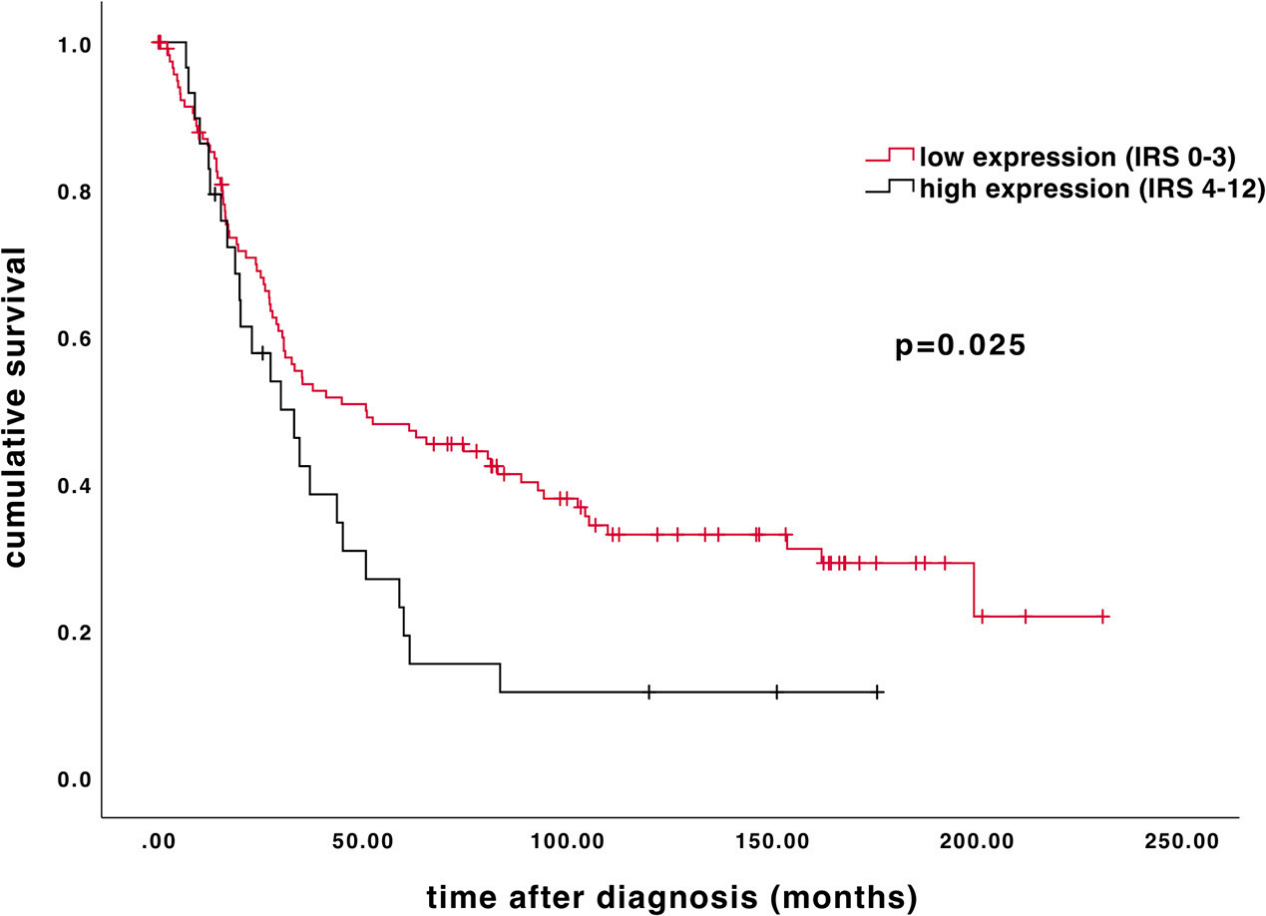
Kaplan–Meier estimate of β-arrestin 2. β-arrestin 2 expression (IRS 4–12) was associated with impaired overall survival (50.64 vs. 29.88 months, median; *p* = 0.025). Censoring events have been marked in the graphs.

Age of the patient cohort was 58.7 ± 31.4 years (median ± standard deviation, SD; *n* = 156) with a range of 31–88 years. The overall survival of the EOC patients was 34.4 ± 57.8 months (median ± SD; *n* = 156). High β-arrestin 2 expression (IRS 4–12) is associated with shorter overall survival. As depicted on the Kaplan-Meier curve, higher cytoplasmic β-arrestin 2 expression correlates with a significantly impaired prognosis (Figure 3; 50.64 vs. 29.88 months, median; *p* = 0.025).

### Clinicopathological Parameters as Independent Prognostic Factors

A multivariate cox-regression analysis was performed to detect which parameters were independent prognostic factors for overall survival in the present cohort. In this analysis, patients' age (*p* = 0.014) and FIGO stage (*p* < 0.001) were independent factors for overall survival. High β-arrestin 2 (*p* = 0.152), however, was not confirmed as an independent factor of prognostic independence (Table 4).

**Table 4:** Multivariate analysis of the analyzed ovarian cancer patients (*n* = 156).

**Covariate**	**Coefficient (b_i_)**	**[HR Exp (b_i_)]**	**95% CI**		***P*-value**
			**Lower**	**Upper**	
Patient's Age (≤ 60 vs. >60)	0.625	1.869	1.133	3.084	0.014
FIGO (I, II vs. III, IV)	0.813	2.255	1.499	3.393	<0.001
**GRADING**					
Clear cell, endometrioid, and mucinous (G1–G3)	0.142	1.153	0.749	1.774	0.517
Serous—low grading	−0.695	0.499	0.160	1.562	0.232
Serous—high grading	0.654	1.924	0.765	4.838	0.164
High β-arrestin 2	−0.097	0.907	0.795	1.036	0.152

### Effects of β-arrestin 2 Overexpression and Depletion on Cell Viability

**Figure 4:**
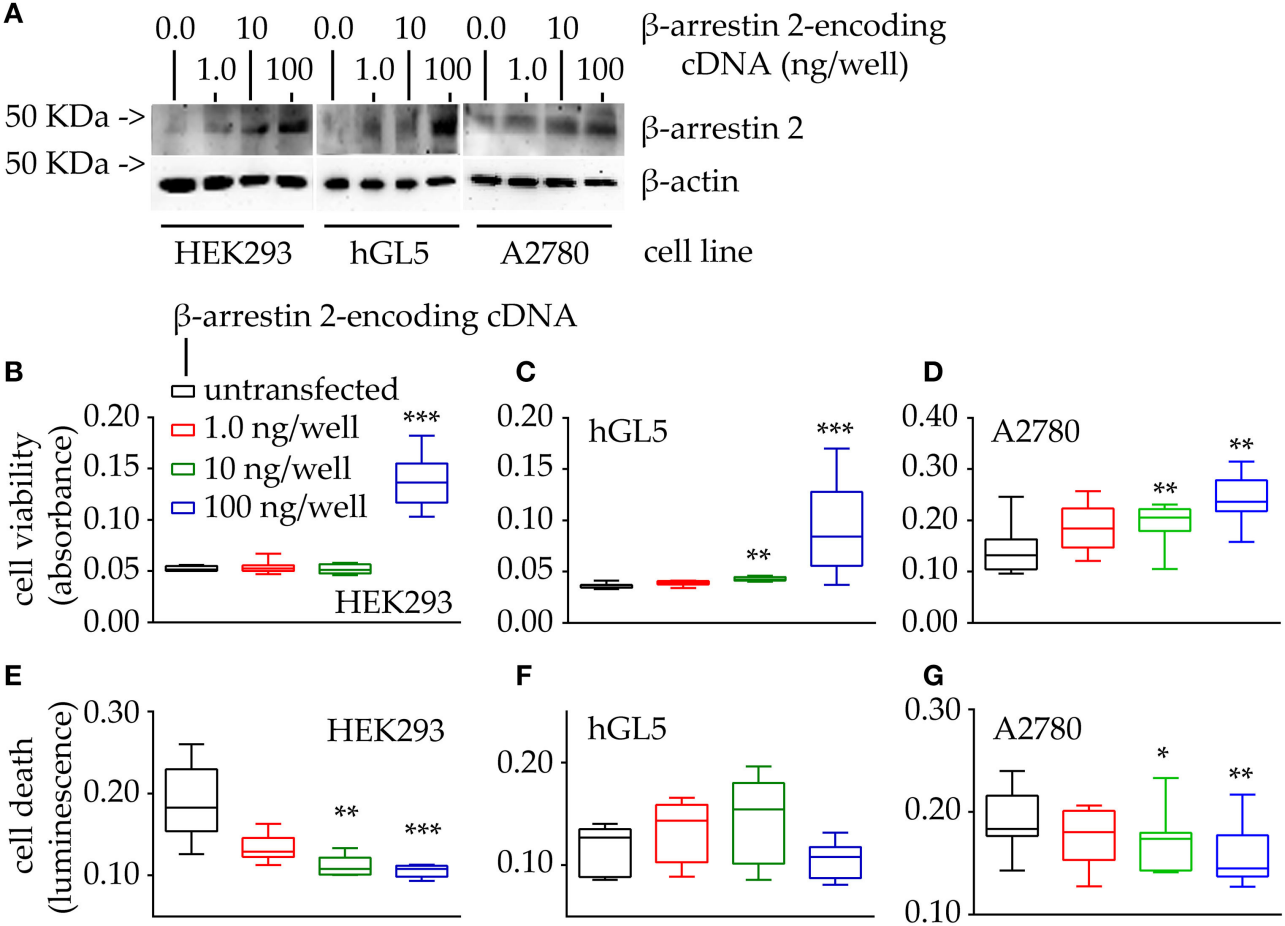
Evaluation of cell viability and death under 48-h β-arrestin 2 overexpression. HEK293, hGL5, and A2780 cells were transfected by increasing amount of plasmid encoding β-arrestin 2 and cell viability and death were assessed by MTT and propidium iodide, respectively. Total protein content from 0.5 × 10^4^ 70% confluent log phase cells were loaded. **(A)** Representative image of β-arrestin 2 detected in cell lysates by Western blotting using a specific rabbit antibody using β-actin as a normalizer. **(B)** Box and Whiskers plot of MTT data, collected from transfected HEK293 cells overexpressing increasing levels of β-arrestin 2, representing cell viability. **(C)** hGL5 cell viability evaluated by MTT. **(D)** β-arrestin 2-dependent cell viability of the A2780 ovarian cancer cell line. **(E)** Data from the β-arrestin 2-overexpressing HEK293, representing cell death, in propidium iodide-stained samples. **(F)** β-arrestin 2-overexpressing hGL5 cell death. **(G)** β-arrestin 2-overexpressing A2780 ovarian cancer cell death. Brightness/contrast of Western blotting pictures were adjusted uniformly in all panels. Asterisks indicate significantly different data distribution than mock-transfected (untransfected) cells (Kruskal-Wallis test and Dunn's correction for multiple comparisons; *P < 0.05; **P < 0.001; ***P < 0.0001; *n* = 8).

Since β-arrestin 2 expression levels were found to correlate with overall survival of ovarian cancer patients, the impact of the molecule on cell proliferation and survival was evaluated, *in vitro*. Cell viability and death are represented by box and whiskers plots (Figure 4).

Western blot analysis demonstrated that β-arrestin 2 expression levels increase together with the amount of cDNA encoding the molecule, in the HEK293, hGL5 and A2780 cell lines (Figure 4A). We found increased viability of all cell models overexpressing β-arrestin 2, compared to mock-transfected cells used as controls (Figures 4B–D; Kruskal-Wallis test; *p* < 0.01; *n* = 8). Moreover, highest levels of cell viability corresponded to the highest amount of plasmid transfected. These results are corroborated by data from propidium iodide-stained HEK293 and A2780 cells overexpressing β-arrestin 2, indicating that the least cell death occurs with the highest amounts of β-arrestin 2-encoding plasmid transfected (Figures 4E–G; Kruskal-Wallis test; *p* < 0.01; *n* = 8). However, data from transfected hGL5 cells did not reveal any association between β-arrestin 2 expression and cell death (Figure 4F; Kruskal-Wallis test; *p* ≥ 0.05; *n* = 8).

**Figure 5:**
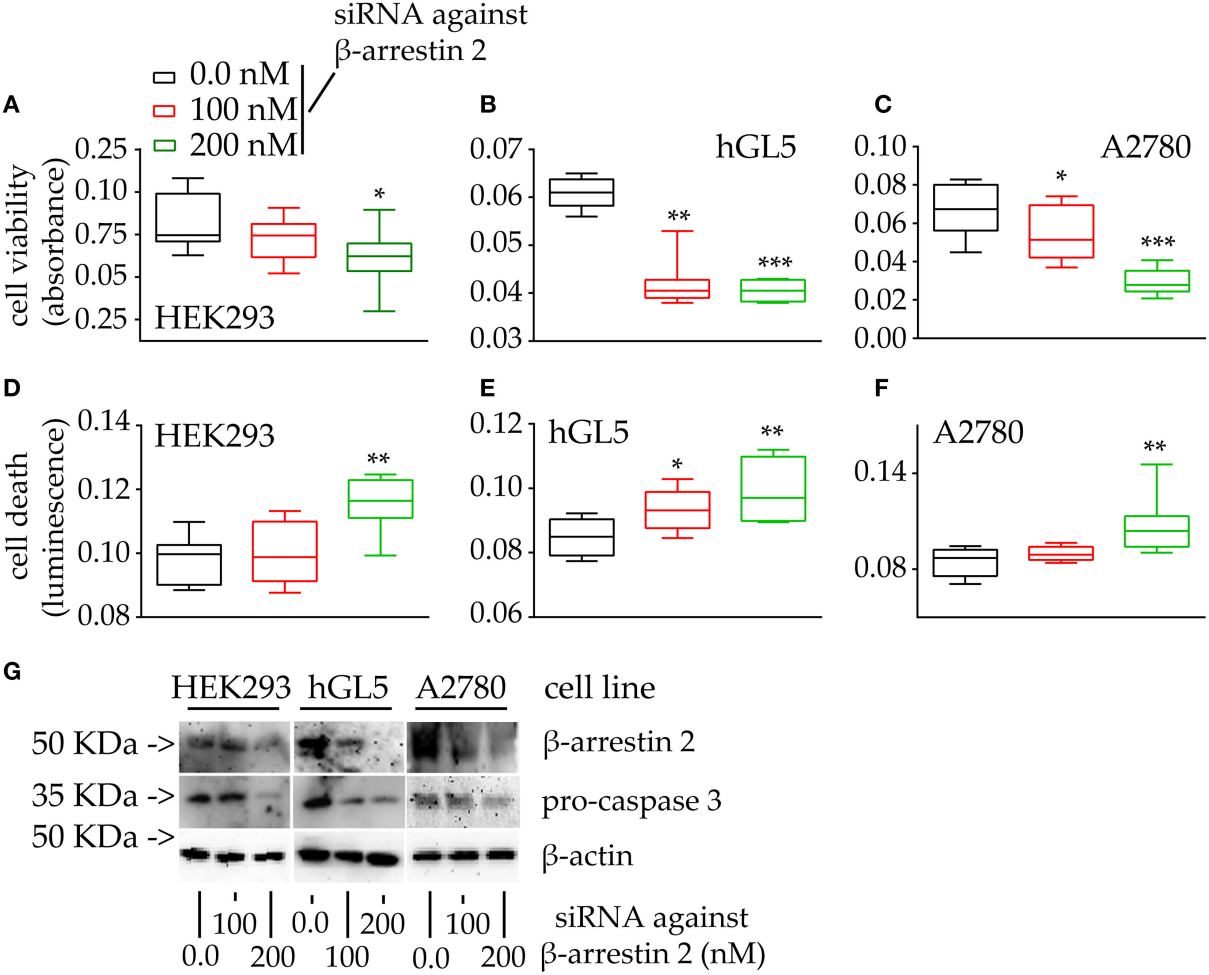
Viability and death of 48-h β-arrestin 2-silenced HEK293, hGL5, and A2780 cells. Samples were transfected by increasing amount of siRNA probes targeting the β-arrestin 2 mRNA and 48-h cell viability and death were assessed by MTT and propidium iodide. Total protein content from 3 × 10^4^ 70% confluent log phase cells were loaded. **(A)** Box and Whiskers plot representing the HEK293 cell viability under β-arrestin 2 depletion. **(B)** β-arrestin 2-silenced hGL5 cell viability. **(C)** β-arrestin 2-silenced A2780 ovarian cancer cell viability. **(D)** Data from the β-arrestin 2-silenced HEK293, representing cell death evaluated using propidium iodide staining. **(E)** hGL5 cell death evaluated by propidium iodide. **(F)** Cell death in β-arrestin 2-silenced A2780 evaluated by propidium iodide. **(G)** Representative Western blotting images demonstrating β-arrestin 2 depletion and pro-caspase 3 cleavage in HEK293, hGL5, and A2780 cell lysates. β-actin was the normalizer. Brightness/contrast of Western blotting pictures were adjusted uniformly in all panels. Significantly different data distribution than mock-transfected (untransfected) cells were indicated by asterisks. Kruskal-Wallis test and Dunn's correction for multiple comparisons; *P < 0.05; **P < 0.001; ***P < 0.0001; *n* = 8).

Since a positive correlation between β-arrestin 2 expression and cell viability was found (Figure 4), control experiments were performed by evaluating the cell viability and death of cells maintained under β-arrestin 2 depletion, *in vitro*. Therefore, HEK293, hGL5, and A2780 cells were treated by increasing concentrations of siRNA probes silencing the endogenous molecule, then MTT assay and propidium iodide staining were performed (Figure 5).

The viability of all the cell lines is significantly reduced upon β-arrestin 2 depletion (Figures 5A–C; Kruskal-Wallis test; *p* < 0.05; *n* = 8). These data are reflected by increased propidium iodide signals occurring together with increasing amounts of siRNAs, in HEK293 cells (Figure 5D; Kruskal-Wallis test; *p* < 0.05; *n* = 8), while similar signals were found between siRNA- and mock-treated hGL5 and A2780 cells (Figures 5E,F; Kruskal-Wallis test; *p* ≥ 0.05; *n* = 8). Western blotting analysis confirmed the siRNA-dependence of β-arrestin 2 silencing, which is linked to pro-caspase 3 cleavage in all the cell lines (Figure 5G).

## Discussion

Our study revealed for the first time that β-arrestin 2 expression significantly correlates with impaired overall survival of ovarian cancer patients and these results are consistent with the proliferative role of β-arrestin 2 demonstrated *in vitro*. β-arrestins are known to act as scaffold proteins controlling multiple cellular functions, such as MAP kinase signaling, GPCR trafficking and transcriptional modulations (38, 39). Several studies demonstrated that β-arrestins are involved in carcinogenesis of the ovary (11, 18) and other types of cancer (10, 12–17). The relevance of the GPCR/β-arrestin pathway in ovarian cancer cells is demonstrated by the finding that endothelin-1 receptor/β-arrestin complex activates beta-catenin signaling pathways with impact on carcinogenesis and metastatic progression (11). Nevertheless, conflicting results have been reported with a positive impact of angiotensin-mediated β-arrestin expression in a clinical-genomic dataset from 820 ovarian cancer patients (40). Indicative results may be provided by the analysis of gene expression data stored in public cancer repositories. For instance, The Human Protein Atlas database (https://www.proteinatlas.org; last accession: 03 March 2020) revealed that β-arrestin 2-encoding gene (*ARRB2*) expression is an unfavorable prognostic marker of prostate cancer, although the association is not replicated for ovarian cancer. These data corroborate, at least in part, our finding although they should be considered cautiously, since gene expression levels are not necessarily corresponding to protein levels and the database does not allow to analyze all the specific histological tumor subtypes.

The link between GPCR/β-arrestins complexes and cancer is an established concept (41) and seems to be result of sustained intracellular signaling occurring upon dysregulation of intracellular β-arrestin protein levels (42). These molecules were associated with drug resistance of breast (43) and lung cancer cells (44), cancer cell migration and invasion (45), tumor progression (46) and metastasis (47), as well as a number of other tumors (48). Thus, β-arrestins were proposed as a target for therapeutic strategies (49). Our data *in vitro* confirm the proliferative role of β-arrestin 2 also in an ovarian cancer cell line, as well as in hGL5 and HEK293 cells. There are converging evidences that β-arrestin 2 depletion leads to decreasing cell viability and/or activating a caspase 3-mediated cell death, at least in HEK293 cells and confirmed in the ovarian cancer cell line A2780. Interestingly, in hGL5 cells, increased β-arrestin 2 expression is linked with upregulation of cell viability, while the depletion of this molecule by siRNA did not result in cell death. This is likely due to the integration of exogenous *E6* and *E7* oncogenes in the hGL5 cell line genome (23), which served for immortalizing them and, likely, results in increased resistance to caspase 3 activation. On the other hand, these oncogenes encode proteins inhibiting tumor protein 53 (p53) and retinoblastoma (Rb) molecules and interfering with apoptosis and cell cycle blockade. hGL5 have characteristics reflecting epithelial tumor cells, such as fibroblast-like spindle shape and relatively high proliferation rate, a feature depending on β-arrestins functioning (21), as well as in other ovarian cancer cell lines (40). However, these results underline that β-arrestins are modulators of proliferative signals exerted through a molecular signaling module with GPCRs and the extracellular-regulated MAP kinases 1 and 2 (ERK1/2), in a number of cells (50), including ovarian cells (21). Gonadotropin receptors are expressed in ovarian cells and targeted by β-arrestin 2 (51). It is well-known that gonadotropin receptor expression is dysregulated in ovarian cancer (34, 36, 52) as well adrenal tumor (53), although the specific influence of these receptor on carcinogenesis is still not determined (54). One reason for this, may be the bi-directional regulation of pro- and anti-apoptotic signals which depend on FSHR and LHCGR expression levels (55). Interestingly, the depletion of β-arrestin 2 may be linked to increased tumor cell growth and angiogenesis, at least in certain cases, such as lung cancer (56). In this case, the absence of β-arrestin 2 would lead to uncontrolled activation of proliferative signals induced by other GPCRs, e.g., the interleukin 8 receptor beta (CXCR2). Taken together, we could speculate that β-arrestins may positively or negatively impact proliferative events, modulating the activity of various GPCRs specifically expressed in the tumor cells.

In correlation analyses, a link between β-arrestin 2 and the G-protein coupled estrogen receptor (GPER) was identified. This receptor is known for rapidly mediating non-genomic signals induced by estrogens and was found in both healthy and neoplastic human tissue (57, 58). Indeed, previous studies revealed that GPER is related to better overall survival in ovarian cancer patients (34, 59, 60). Since GPER belongs to the GPCRs family, we may suppose that β-arrestin 2 might interact with this membrane estrogen receptor and influence its physiological function. However, this issue must be further investigated because GPER recycling and intracellular trafficking seems to be regulated by mechanisms not involving β-arrestins (61).

Interestingly, we also found an association between β-arrestin 2 and one of the two LHCGR ligands, hCGβ. This hormone was shown to be associated with an increased 5-years survival in LHCGR positive/FSHR negative ovarian cancer cases (33). Although this study had limitations related to low specificity of the anti-gonadotropin receptor antibodies employed (62), it suggests that hCGβ and its receptor might be linked to survival in these patients. These data would be in line with the relatively high potency of hCGβ in inducing LHCGR-mediated intracellular cAMP increase (27), as an effect linked to the steroidogenic potential of the hormone and to pro-apoptotic effects (24). On the other hand, these data would be in contrast with the fact that hCGβ has been used as a marker of certain tumors, such as the choriocarcinoma (63), which reflects the involvement of β-arrestin 2 in the hCGβ-mediated intracellular signaling (29) and suggests that the connection between the two molecules in cancer merits further investigations.

β-arrestin 2 is associated with a significantly impaired overall survival of ovarian cancer patients. This finding is supported by *in vitro* data demonstrating that β-arrestin 2 upregulates the viability of an ovarian cancer cell line, and transfected HEK293 and hGL5 cells. Reflecting the role of β-arrestins as scaffold proteins linked to the GPCRs activity, correlations between the β-arrestin 2 and FSHR and LHCGR expression was identified ovarian cancer tissue. Taken together, we indicate that β-arrestin 2 may be a promising new target in ovarian cancer so that clinical implications should be further addressed in future research.

## Data Availability Statement

The raw data supporting the conclusions of this article will be made available by the authors, without undue reservation.

## Ethics Statement

The studies involving human participants were reviewed and approved by Ethics Committee of the Ludwig-Maximilians-University, Munich, Germany. Written informed consent for participation was not required for this study in accordance with the national legislation and the institutional requirements.

## Author Contributions

BC, UJ, MS, LC, and FT: conceptualization. DM, SM, and AB: validation. BC, AP, UJ, and LC: formal analysis. AP, BM, RB, and SB: investigation. BC, AP, LC, and FT: writing-original draft preparation. UJ, VS, DM, SM, AB, MS, BM, RB, and SB: writing—review and editing. AP, BC, and LC: visualization. UJ, SM, and MS: supervision. All authors have read and agreed to the published version of the manuscript.

## Conflict of Interest

Research support, advisory board, honoraria, and travel expenses from AstraZeneca, Clovis, Medac, MSD, Novartis, PharmaMar, Roche, Sensor Kinesis, Tesaro, Teva have been received by SM and from AstraZeneca, Medac, PharmaMar, Roche, Tesaro/GSK by FT. All above mentioned companies did not influence the study design, data collection and analysis, decision to publish, or preparation of the manuscript. The remaining authors declare that the research was conducted in the absence of any commercial or financial relationships that could be construed as a potential conflict of interest.

## References

[1] Siegel RL Miller KD Jemal A Cancer statistics 2019. CA Cancer J Clin. (2019). 69:7–34. 10.3322/caac.21551 30620402

[2] Baldwin LA Huang B Miller RW Tucker T Goodrich ST Podzielinski I. Ten-year relative survival for epithelial ovarian cancer. Obstet Gynecol. (2012) 120:612–8. 10.1097/AOG.0b013e318264f794 22914471

[3] du Bois A Reuss A Pujade-Lauraine E Harter P Ray-Coquard I Pfisterer J Role of surgical outcome as prognostic factor in advanced epithelial ovarian cancer: a combined exploratory analysis of 3 prospectively randomized phase 3 multicenter trials: by the arbeitsgemeinschaft gynaekologische onkologie studiengruppe ovarialkarzinom (AGO-OVAR) and the groupe d'investigateurs nationaux pour les etudes des cancers de l'Ovaire (GINECO). Cancer. (2009) 115:1234–44. 10.1002/cncr.24149 19189349

[4] Aletti GD Gostout BS Podratz KC Cliby WA. Ovarian cancer surgical resectability: relative impact of disease, patient status, and surgeon. Gynecol Oncol. (2006) 100:33–7. 10.1016/j.ygyno.2005.07.123 16153692

[5] Vergote I De Brabanter J Fyles A Bertelsen K Einhorn N Sevelda P. Prognostic importance of degree of differentiation and cyst rupture in stage I invasive epithelial ovarian carcinoma. Lancet. (2001) 357:176–82. 10.1016/S0140-6736(00)03590-X 11213094

[6] Dembo AJ Davy M Stenwig AE Berle EJ Bush RS Kjorstad KK Prognostic factors in patients with stage I epithelial ovarian cancer. Obstet Gynecol. (1990) 75:263–73. 2300355

[7] Tomao F Marchetti C Romito A Di Pinto A Di Donato V Capri O. Overcoming platinum resistance in ovarian cancer treatment: from clinical practice to emerging chemical therapies. Expert Opin Pharmacother. (2017) 18:1443–55. 10.1080/14656566.2017.1328055 28521614

[8] Kossai M Leary A Scoazec JY Genestie C. Ovarian cancer: a heterogeneous disease. Pathobiology. (2018) 85:41–9. 10.1159/000479006 29020678

[9] Shenoy SK Lefkowitz RJ. β-arrestin-mediated receptor trafficking and signal transduction. Trends Pharmacol Sci. (2011) 32:521–33. 10.1016/j.tips.2011.05.002 21680031 PMC3159699

[10] Mythreye K Blobe GC. The type III TGF-beta receptor regulates epithelial and cancer cell migration through beta-arrestin2-mediated activation of Cdc42. Proc Natl Acad Sci USA. (2009) 106:8221–6. 10.1073/pnas.0812879106 19416857 PMC2688894

[11] Rosano L Cianfrocca R Masi S Spinella F Di Castro V Biroccio A. Beta-arrestin links endothelin A receptor to beta-catenin signaling to induce ovarian cancer cell invasion and metastasis. Proc Natl Acad Sci USA. (2009) 106:2806–11. 10.1073/pnas.0807158106 19202075 PMC2650347

[12] Alvarez CJP Lodeiro M Theodoropoulou M Camina JP Casanueva FF Pazos Y. Obestatin stimulates Akt signalling in gastric cancer cells through beta-arrestin-mediated epidermal growth factor receptor transactivation. Endocr Relat Cancer. (2009) 16:599–611. 10.1677/ERC-08-0192 19153210

[13] Dasgupta P Rizwani W Pillai S Davis R Banerjee S Hug K. ARRB1-mediated regulation of E2F target genes in nicotine-induced growth of lung tumors. J Natl Cancer Inst. (2011) 103:317–33. 10.1093/jnci/djq541 21212384 PMC3039728

[14] Buchanan FG Gorden DL Matta P Shi Q Matrisian LM DuBois RN. Role of beta-arrestin 1 in the metastatic progression of colorectal cancer. Proc Natl Acad Sci USA. (2006) 103:1492–7. 10.1073/pnas.0510562103 16432186 PMC1360588

[15] Moussa O Ashton AW Fraig M Garrett-Mayer E Ghoneim MA Halushka PV. Novel role of thromboxane receptors beta isoform in bladder cancer pathogenesis. Cancer Res. (2008) 68:4097–104. 10.1158/0008-5472.CAN-07-6560 18519668

[16] Li TT Alemayehu M Aziziyeh AI Pape C Pampillo M Postovit LM. Beta-arrestin/Ral signaling regulates lysophosphatidic acid-mediated migration and invasion of human breast tumor cells. Mol Cancer Res. (2009) 7:1064–77. 10.1158/1541-7786.MCR-08-0578 19609003

[17] Lin HK Wang L Hu YC Altuwaijri S Chang C. Phosphorylation-dependent ubiquitylation and degradation of androgen receptor by Akt require Mdm2 E3 ligase. EMBO J. (2002) 21:4037–48. 10.1093/emboj/cdf406 12145204 PMC126152

[18] Rosano L Spinella F Di Castro V Nicotra MR Dedhar S de Herreros AG. Endothelin-1 promotes epithelial-to-mesenchymal transition in human ovarian cancer cells. Cancer Res. (2005) 65:11649–57. 10.1158/0008-5472.CAN-05-2123 16357176

[19] Riccetti L Klett D Ayoub MA Boulo T Pignatti E Tagliavini S. Heterogeneous hCG and hMG commercial preparations result in different intracellular signalling but induce a similar long-term progesterone response *in vitro*. Mol Hum Reprod. (2017) 23:685–97. 10.1093/molehr/gax047 29044421

[20] Riccetti L Sperduti S Lazzaretti C Klett D De Pascali F Paradiso E. Glycosylation pattern and *in vitro* bioactivity of reference follitropin alfa and biosimilars. Front Endocrinol. (2019) 10:503. 10.3389/fendo.2019.00503 31396162 PMC6667556

[21] Casarini L Reiter E Simoni M β-arrestins regulate gonadotropin receptor-mediated cell proliferation and apoptosis by controlling different FSHR or LHCGR intracellular signaling in the hGL5 cell line. Mol Cell Endocrinol. (2016) 437:11–21. 10.1016/j.mce.2016.08.005 27502035

[22] Liu J Li QX Wang XJ Zhang C Duan YQ Wang ZY. β-Arrestins promote podocyte injury by inhibition of autophagy in diabetic nephropathy. Cell Death Dis. (2016) 7:e2183. 10.1038/cddis.2016.89 27054338 PMC4855668

[23] Rainey WH Sawetawan C Shay JW Michael MD Mathis JM Kutteh W. Transformation of human granulosa cells with the E6 and E7 regions of human papillomavirus. J Clin Endocrinol Metab. (1994) 78:705–10. 10.1210/jcem.78.3.8126145 8126145

[24] Casarini L Riccetti L De Pascali F Gilioli L Marino M Vecchi E. Estrogen modulates specific life and death signals induced by LH and hCG in human primary granulosa cells *in vitro*. Int J Mol Sci. (2017) 18:926. 10.3390/ijms18050926 28452938 PMC5454839

[25] Sperduti S Limoncella S Lazzaretti C Paradiso E Riccetti L Turchi S. GnRH antagonists produce differential modulation of the signaling pathways mediated by GnRH receptors. Int J Mol Sci. (2019) 20:5548. 10.3390/ijms20225548 31703269 PMC6888270

[26] Lazzaretti C Riccetti L Sperduti S Anzivino C Brigante G De Pascali F. Inferring biallelism of two FSH receptor mutations associated with spontaneous ovarian hyperstimulation syndrome by evaluating FSH, LH and HCG cross-activity. Reprod Biomed Online. (2019) 38:816–24. 10.1016/j.rbmo.2018.12.021 30910395

[27] Casarini L Riccetti L Limoncella S Lazzaretti C Barbagallo F Pacifico S. Probing the effect of sildenafil on progesterone and testosterone production by an intracellular FRET/BRET combined approach. Biochemistry. (2019) 58:799–808. 10.1021/acs.biochem.8b01073 30532959

[28] Brigante G Riccetti L Lazzaretti C Rofrano L Sperduti S Poti F. Abacavir, nevirapine, and ritonavir modulate intracellular calcium levels without affecting GHRH-mediated growth hormone secretion in somatotropic cells *in vitro*. Mol Cell Endocrinol. (2019) 482:37–44. 10.1016/j.mce.2018.12.005 30543878

[29] Riccetti L Yvinec R Klett D Gallay N Combarnous Y Reiter E. Human luteinizing hormone and chorionic gonadotropin display biased agonism at the LH and LH/CG receptors. Sci Rep. (2017) 7:940. 10.1038/s41598-017-01078-8 28424471 PMC5430435

[30] Casarini L Lispi M Longobardi S Milosa F La Marca A Tagliasacchi D. LH and hCG action on the same receptor results in quantitatively and qualitatively different intracellular signalling. PLoS ONE. (2012) 7:e46682. 10.1371/journal.pone.0046682 23071612 PMC3465272

[31] Mosmann T. Rapid colorimetric assay for cellular growth and survival: application to proliferation and cytotoxicity assays. J Immunol Methods. (1983) 65:55–63. 10.1016/0022-1759(83)90303-4 6606682

[32] Kabakov AE Gabai VL. Cell death survival assays. Methods Mol Biol. (2018) 1709:107–27. 10.1007/978-1-4939-7477-1_9 29177655

[33] Lenhard M Tsvilina A Schumacher L Kupka M Ditsch N Mayr D. Human chorionic gonadotropin and its relation to grade, stage and patient survival in ovarian cancer. BMC Cancer. (2012) 12:2. 10.1186/1471-2407-12-2 22214378 PMC3311592

[34] Heublein S Mayr D Vrekoussis T Friese K Hofmann SS Jeschke U. The G-protein coupled estrogen receptor (GPER/GPR30) is a gonadotropin receptor dependent positive prognosticator in ovarian carcinoma patients. PLoS ONE. (2013) 8:e71791. 10.1371/journal.pone.0071791 23951246 PMC3739730

[35] Heublein S Vrekoussis T Mayr D Friese K Lenhard M Jeschke U. Her-2/neu expression is a negative prognosticator in ovarian cancer cases that do not express the follicle stimulating hormone receptor (FSHR). J Ovarian Res. (2013) 6:6. 10.1186/1757-2215-6-6 23339713 PMC3557169

[36] Scholz C Heublein S Lenhard M Friese K Mayr D Jeschke U. Glycodelin A is a prognostic marker to predict poor outcome in advanced stage ovarian cancer patients. BMC Res Notes. (2012) 5:551. 10.1186/1756-0500-5-551 23036050 PMC3599868

[37] Deuster E Mayr D Hester A Kolben T Zeder-Goss C Burges A. Correlation of the aryl hydrocarbon receptor with FSHR in ovarian cancer patients. Int J Mol Sci. (2019) 20:2862. 10.3390/ijms20122862 31212758 PMC6628023

[38] DeWire SM Ahn S Lefkowitz RJ Shenoy SK. Beta-arrestins cell signaling. Annu Rev Physiol. (2007) 69:483–510. 10.1146/annurev.physiol.69.022405.154749 17305471

[39] Kang DS Tian X Benovic JL. Role of beta-arrestins and arrestin domain-containing proteins in G protein-coupled receptor trafficking. Curr Opin Cell Biol. (2014) 27:63–71. 10.1016/j.ceb.2013.11.005 24680432 PMC3971387

[40] Bush SH Tollin S Marchion DC Xiong Y Abbasi F Ramirez IJ. Sensitivity of ovarian cancer cells to acetaminophen reveals biological pathways that affect patient survival. Mol Clin Oncol. (2016) 4:399–404. 10.3892/mco.2016.725 26998291 PMC4774474

[41] Suleymanova N Crudden C Shibano T Worrall C Oprea I Tica A. Functional antagonism of beta-arrestin isoforms balance IGF-1R expression and signalling with distinct cancer-related biological outcomes. Oncogene. (2017) 36:5734–44. 10.1038/onc.2017.179 28581517 PMC5658667

[42] Thomsen ARB Plouffe B Cahill TJ III Shukla AK Tarrasch JT Dosey AM. GPCR-G protein-beta-arrestin super-complex mediates sustained G protein signaling. Cell. (2016) 166:907–19. 10.1016/j.cell.2016.07.004 27499021 PMC5418658

[43] Jing X Zhang H Hu J Su P Zhang W Jia M. β-arrestin 2 is associated with multidrug resistance in breast cancer cells through regulating MDR1 gene expression. Int J Clin Exp Pathol. (2015) 8:1354–63. 25973019 PMC4396277

[44] Becker JH Gao Y Soucheray M Pulido I Kikuchi E Rodriguez ML. CXCR7 reactivates ERK signaling to promote resistance to EGFR kinase inhibitors in NSCLC. Cancer Res. (2019) 79:4439–52. 10.1158/0008-5472.CAN-19-0024 31273063 PMC6746175

[45] Rosano L Bagnato A. New insights into the regulation of the actin cytoskeleton dynamics by GPCR/beta-arrestin in cancer invasion and metastasis. Int Rev Cell Mol Biol. (2019) 346:129–55. 10.1016/bs.ircmb.2019.03.002 31122393

[46] Son D Kim Y Lim S Kang HG Kim DH Park JW. miR-374a-5p promotes tumor progression by targeting ARRB1 in triple negative breast cancer. Cancer Lett. (2019) 454:224–33. 10.1016/j.canlet.2019.04.006 31004703

[47] Ji H Liu N Li J Chen D Luo D Sun Q. Oxytocin involves in chronic stress-evoked melanoma metastasis via beta-arrestin 2-mediated ERK signaling pathway. Carcinogenesis. (2019) 40:1395–404. 10.1093/carcin/bgz064 30923807

[48] Song Q Ji Q Li Q. The role and mechanism of betaarrestins in cancer invasion and metastasis. Int J Mol Med. (2018) 41:631–9. 10.3892/ijmm.2017.3288 29207104 PMC5752234

[49] Peterson YK Luttrell LM. The diverse roles of arrestin scaffolds in G protein-coupled receptor signaling. Pharmacol Rev. (2017) 69:256–97. 10.1124/pr.116.013367 28626043 PMC5482185

[50] Carmona-Rosas G Alcantara-Hernandez R Hernandez-Espinosa DA. Dissecting the signaling features of the multi-protein complex GPCR/beta-arrestin/ERK1/2. Eur J Cell Biol. (2018) 97:349–58. 10.1016/j.ejcb.2018.04.001 29665971

[51] Ayoub MA Landomiel F Gallay N Jegot G Poupon A Crepieux P. Assessing gonadotropin receptor function by resonance energy transfer-based assays. Front Endocrinol. (2015) 6:130. 10.3389/fendo.2015.00130 26379624 PMC4550792

[52] Feng Z Wen H Bi R Ju X Chen X Yang W. A clinically applicable molecular classification for high-grade serous ovarian cancer based on hormone receptor expression. Sci Rep. (2016) 6:25408. 10.1038/srep25408 27139372 PMC4853732

[53] Bernichtein S Peltoketo H Huhtaniemi I. Adrenal hyperplasia and tumours in mice in connection with aberrant pituitary-gonadal function. Mol Cell Endocrinol. (2009) 300:164–8. 10.1016/j.mce.2008.10.005 19007852

[54] Nordhoff V Gromoll J Simoni M. Constitutively active mutations of G protein-coupled receptors: the case of the human luteinizing hormone and follicle-stimulating hormone receptors. Arch Med Res. (1999) 30:501–9. 10.1016/S0188-4409(99)00076-4 10714364

[55] Casarini L Santi D Simoni M Poti F. 'Spare' luteinizing hormone receptors: facts and fiction. Trends Endocrinol Metab. (2018) 29:208–17. 10.1016/j.tem.2018.01.007 29429918

[56] Raghuwanshi SK Nasser MW Chen X Strieter RM Richardson RM. Depletion of beta-arrestin-2 promotes tumor growth and angiogenesis in a murine model of lung cancer. J Immunol. (2008) 180:5699–706. 10.4049/jimmunol.180.8.5699 18390755

[57] Jala VR Radde BN Haribabu B Klinge CM. Enhanced expression of G-protein coupled estrogen receptor (GPER/GPR30) in lung cancer. BMC Cancer. (2012) 12:624. 10.1186/1471-2407-12-624 23273253 PMC3557142

[58] Ignatov A Ignatov T Weissenborn C Eggemann H Bischoff J Semczuk A. G-protein-coupled estrogen receptor GPR30 and tamoxifen resistance in breast cancer. Breast Cancer Res Treat. (2011) 128:457–66. 10.1007/s10549-011-1584-1 21607586

[59] Heublein S Lenhard M Vrekoussis T Schoepfer J Kuhn C Friese K. The G-protein-coupled estrogen receptor (GPER) is expressed in normal human ovaries and is upregulated in ovarian endometriosis and pelvic inflammatory disease involving the ovary. Reprod Sci. (2012) 19:1197–204. 10.1177/1933719112446085 22573494

[60] Ignatov T Modl S Thulig M Weissenborn C Treeck O Ortmann O. GPER-1 acts as a tumor suppressor in ovarian cancer. J Ovarian Res. (2013) 6:51. 10.1186/1757-2215-6-51 23849542 PMC3723961

[61] Cheng SB Quinn JA Graeber CT Filardo EJ. Down-modulation of the G-protein-coupled estrogen receptor, GPER, from the cell surface occurs via a trans-Golgi-proteasome pathway. J Biol Chem. (2011) 286:22441–55. 10.1074/jbc.M111.224071 21540189 PMC3121390

[62] Chrusciel M Ponikwicka-Tyszko D Wolczynski S Huhtaniemi I Rahman NA. Extragonadal FSHR expression and function-is it real? Front Endocrinol. (2019) 10:32. 10.3389/fendo.2019.00032 30778333 PMC6369633

[63] Cole LA Hartle RJ Laferla JJ Ruddon RW. Detection of the free beta subunit of human chorionic gonadotropin (HCG) in cultures of normal and malignant trophoblast cells, pregnancy sera, and sera of patients with choriocarcinoma. Endocrinology. (1983) 113:1176–8. 10.1210/endo-113-3-1176 6191970

