# Supplementary material for: β-arrestin 2 Is a Prognostic Factor for Survival of Ovarian Cancer Patients Upregulating Cell Proliferation
Source: Front Endocrinol (Lausanne). 2020 Sep 18;11:554733. doi: 10.3389/fendo.2020.554733 (PMC7530235; doi:10.3389/fendo.2020.554733)
Supplement: Supplementary file 1 [file Image_1.pdf]

Supplementary Material

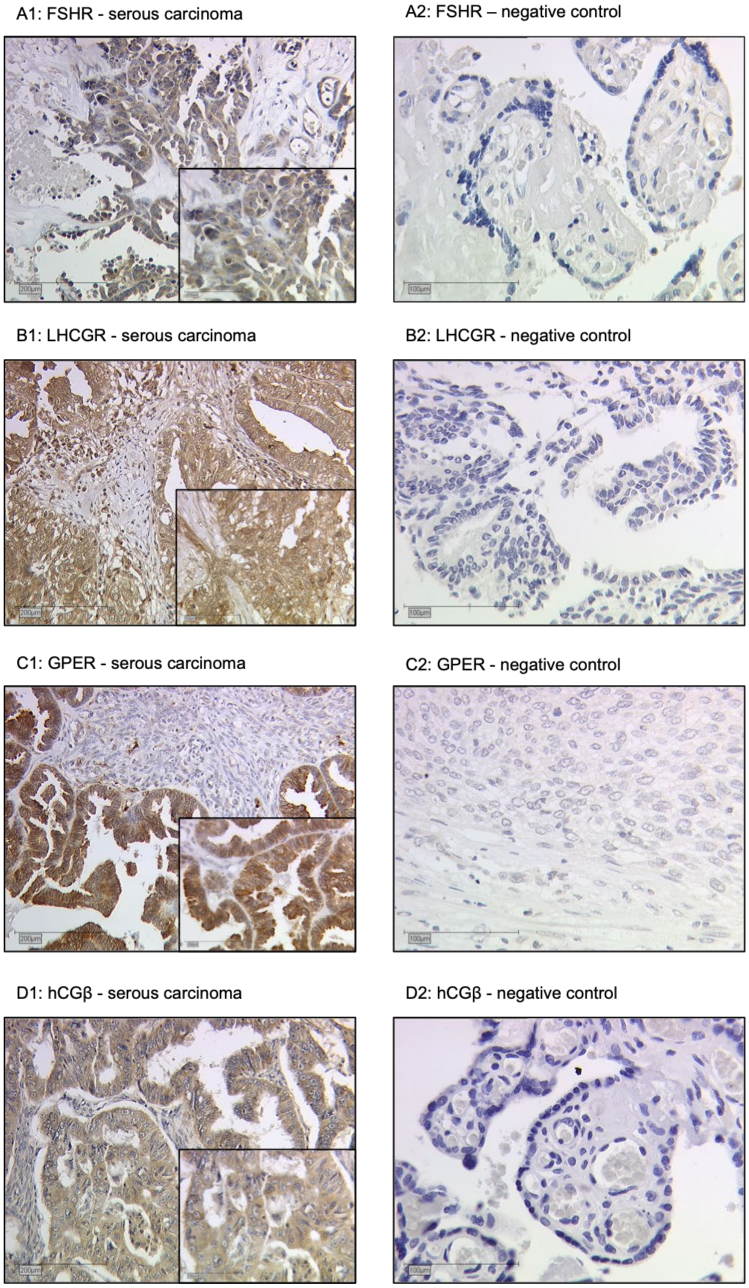

Supplementary Figure 1. Detection of FSHR, LHCGR, GPER and hCGβ with immunohistochemistry. (A1) FSHR, (B1) LHCGR, (C1) GPER, (D1) and hCGβ in serous ovarian cancer tissue with the corresponding negative controls (A2-D2).
